# Examining the Effectiveness of 3D Virtual Reality Training on Problem-solving, Self-efficacy, and Teamwork Among Inexperienced Volunteers Helping With Drug Use Prevention: Randomized Controlled Trial

**DOI:** 10.2196/29862

**Published:** 2021-11-02

**Authors:** Chih-Huei Chiang, Chiu-Mieh Huang, Jiunn-Jye Sheu, Jung-Yu Liao, Hsiao-Pei Hsu, Shih-Wen Wang, Jong-Long Guo

**Affiliations:** 1 Department of Health Promotion and Health Education, College of Education National Taiwan Normal University Taipei Taiwan; 2 Institute of Clinical Nursing, College of Nursing National Yang Ming Chiao Tung University Taipei Taiwan; 3 School of Population Health University of Toledo Toledo, OH United States; 4 Department of Public Health Kaohsiung Medical University Kaohsiung Taiwan; 5 Department of Nursing, College of Nursing National Yang Ming Chiao Tung University Taipei Taiwan

**Keywords:** 3D virtual reality, volunteers, problem-solving, self-efficacy, teamwork

## Abstract

**Background:**

Illegal drug usage among adolescents is a critical health problem. The Taiwanese government provides an accompanying volunteer program to prevent students who experiment with drugs from reusing them. An appropriate training program can improve volunteers’ abilities to assist students using drugs. Problem-solving, self-efficacy, and teamwork are critical abilities for inexperienced volunteers who help with drug use prevention. By interacting with the animation or 3D virtual reality (VR) in the virtual scene, learners can immerse themselves in the virtual environment to learn, and 3D VR can increase learning opportunities and reduce the cost of human and material resources.

**Objective:**

The aim of this study was to examine the effectiveness of spherical video-based virtual reality (SVVR) training in improving problem-solving, self-efficacy, and teamwork among volunteers who helped prevent adolescents from using illegal drugs.

**Methods:**

This study used a randomized controlled design with a total of 68 participants in the experimental (n=35) and control (n=33) groups. The participants in the experimental group received the SVVR training program and their counterparts in the control group did not receive any training.

**Results:**

Generalized estimating equation analyses indicated that the experimental group showed significant posttraining improvements in problem-solving and self-efficacy but not teamwork when compared with the control group.

**Conclusions:**

The results of this study revealed that SVVR could improve participants’ problem-solving skills and self-efficacy for assisting students in not using illegal drugs. However, future studies are suggested to develop effective SVVR to assist inexperienced volunteers in enhancing their teamwork abilities. We believed that introducing the training program to more sites can enhance volunteer training so that volunteers can have a better companionship effect when helping students quit drugs.

**Trial Registration:**

ClinicalTrials.gov NCT05072431; https://clinicaltrials.gov/ct2/show/NCT05072431

## Introduction

Illegal drugs contain psychoactive substances and toxic chemicals that are harmful to adolescents. Numerous countries ban the use of illegal drugs by adolescents because it is highly associated with poor brain function [1], cognitive abilities [2], and academic performance [2]. In addition, drug use during adolescence predicts future drug-related disorders in adulthood [3]. Since recent years, new psychoactive substances are being widely used by adolescents in many countries, causing serious health consequences [4]. Thus, prevention of illegal drug and new psychoactive substance usage has become a critical and urgent issue for governments and schools worldwide.

The Taiwanese government is fully aware of the seriousness of adolescent drug use. In addition to setting up an intervention program to prevent students who experiment with drugs from reusing them [5], the Taiwanese government also provides an accompanying volunteer program (ie, the Sunshine volunteer program). To be a volunteer, an individual is expected to be enthusiastic and willing to be a life coach to adolescents with drug use history and assist them in quitting drugs. Before serving, volunteers are required to complete 12 hours each of basic and specific training on drug use prevention [6]. Currently, the 12-hour training program involves a lecture delivered using the traditional teaching method. Informed by a needs assessment before the study inception, volunteers told us that they would like to learn problem-solving skills and increase their self-efficacy to meet the job requirements when supporting students in not using illegal drugs. In addition, inexperienced volunteers would like to form good teams with students to work together in preventing drug use. Although the existing training program can increase professional knowledge on drug use prevention, it is insufficient in the aspects of problem-solving abilities, self-efficacy, and teamwork skills. Thus, it is critical to develop an innovative training program.

An appropriate training program can improve problem-solving abilities among volunteers [7]. Problem-solving pedagogy can assist learners to use existing knowledge as well as previous experiences and skills to generate effective and feasible solutions to present problems [8]. Therefore, the problem-solving teaching method is important to cultivate learners’ abilities [9,10].

Self-efficacy is a critical construct of the social cognitive theory advocated by Bandura [11]. Self-efficacy refers to an individual's belief that he or she can perform the necessary behaviors to produce specific performance achievements. Self-efficacy reflects self-confidence in one's motivation, behavior, and social environment. These cognitive self-assessments influence all types of human experiences, including the goals that people strive for, energy they spend to achieve them, and possibility of reaching a certain level of behavioral performance [12]. The construct of self-efficacy has been widely employed in drug use prevention. For example, a previous study applying an electronic course program on drug use prevention indicated that the adolescent participants in the experimental group displayed better self-efficacy in terms of drug use resistance than their counterparts in the comparison group [5].

To help students stop using illegal drugs, teamwork is important for volunteers. Teamwork might include team building and collaborative discussions of the adolescents’ daily life. A previous study indicated that teamwork might buffer the negative effects of the drinking environment on coworkers [13]. Moreover, a previous study indicated that teamwork is an essential component of a preventive program to help manage problem behaviors [14]. A proposed model also suggested that better teamwork among parenting partners might reduce an important set of family risk factors associated with drug use as well as behavioral and emotional problems in children [15].

Problem-solving, self-efficacy, and teamwork are critical abilities for inexperienced volunteers who help with drug use prevention. Lectures are the main components of the traditional training programs, which can be replaced by integrating with emerging technology. A virtual learning environment is provided by 3D virtual reality (3D VR). By interacting with the animation or 3D VR in the virtual scene, learners can immerse themselves in the virtual environment to learn, and 3D VR can increase learning opportunities and reduce the cost of human and material resources. Empirical research indicates that VR-based teaching can provide participants with new experiences and increase their learning ability [16].

The use of 3D VR with multiple perspectives and multisensory cues offers several potential benefits to education and training [17]. Furthermore, 3D VR promotes experiential and active learning, visualization and reification, learning in contexts impossible or difficult to experience in real life, motivation enhancement, collaboration fostering, adaptability, and evaluation and assessment. Previous studies also documented 3D VR as a useful intervention tool to train older adults [18,19]. Considering the success reported in the literature, this study projected the likelihood of the training effects on adult volunteers who helped prevent illegal drug use.

The 360-degree view Camera (VR Camera) is a useful hardware device to provide VR content. The VR-based 360-degree scenery video is a combination of image computing technology and VR wearables that allow learners to obtain an immersive experience. In recent years, VR techniques and spherical video-based virtual reality (SVVR) have made significant progress in numerous training programs, allowing learners to interact with the virtual world. For example, researchers applied this technology to train people with public speaking anxiety to improve their performance. The results showed that participants with moderate and high levels of speaking anxiety did show improvements after receiving training [20]. In addition, a previous study also showed that SVVR-based learning is effective for childbirth training in nursing education. These findings encouraged the use of SVVR-based training programs [21].

Researchers also applied VR technology in clinical settings and explored its effectiveness in the field of substance use. For example, VR technology and cue responses were used to explore smokers' cravings for smoking [22]. Saladin et al [23] found that 3D VR could induce physiological and psychological responses, and then produce the intention and behavior related to drug use. A previous study used VR to assist smokers in quitting smoking. It found that crushing cigarettes significantly improved the score of nicotine addiction and the rate of quitting smoking, as well as reduced the loss of patients [24].

Another advantage of VR-based education is that such interactive training is more suitable for sensitive problems, such as drug use, to avoid ethical disputes. For example, it may not be appropriate to provide real drugs at the teaching site; however, virtual drugs can be allowed in a VR environment. To the best of our knowledge, this SVVR-based training is the first of its kind for volunteers. This study aims to develop an SVVR-based training program for volunteers helping in preventing illegal drug use by adolescents and evaluate the effectiveness of the training program.

## Methods

### Participants

Sunshine volunteers helped students with illegal drug use problem in schools. Each city and county had a volunteer group. After we sent an invitation letter to each of the 17 Sunshine volunteer groups, 5 of them agreed to participate. We invited all volunteers in the 5 groups (approximately 200) to participate in our study and randomized them into the experimental and control groups by assigning a number and flipping a coin. The selection criteria of participants were (1) helping at least 1 student with drug use problems, (2) having served for less than 2 years as a Sunshine volunteer, (3) having experience in using technology products, and (4) being able to operate SVVR. Recognizing service experience as a confounding factor in our sampling, we selected only the volunteers with less than 2 years of experience to minimize its effect. Training these novice volunteers was expected to produce greater improvements than enlisting experienced volunteers. There was no cybersickness incident that led to the exclusion of participants. [25]. After provision of their signed informed consent forms, 68 selected volunteers participated in the study and were allocated randomly into experimental and control groups by tossing a coin, leading to 35 and 33 participants in the experimental and control groups, respectively.

The participants in the control group received the SVVR intervention after completion of the study. The research team conducted one-on-one interviews to obtain the survey data. All participants successfully completed the program within approximately 3 hours.

According to Kirk [26], for an estimated effect size of 0.80, the approximate sample size required is 26 for each group when the power is set at 0.80 and type I error at 0.05. A previous 3D VR study yielded significant preintervention and postintervention improvements in psychological health with a similar sample size [19]. Another SVVR study with 32 participants in the experimental and control groups showed that providing childbirth education with SVVR resulted in superior learning performance [21]. Therefore, the sample size in this study (N=68) was large enough to detect training effects. Moreover, all participants finished the training program successfully.

### Recruitment and Assessments

**Figure 1:**
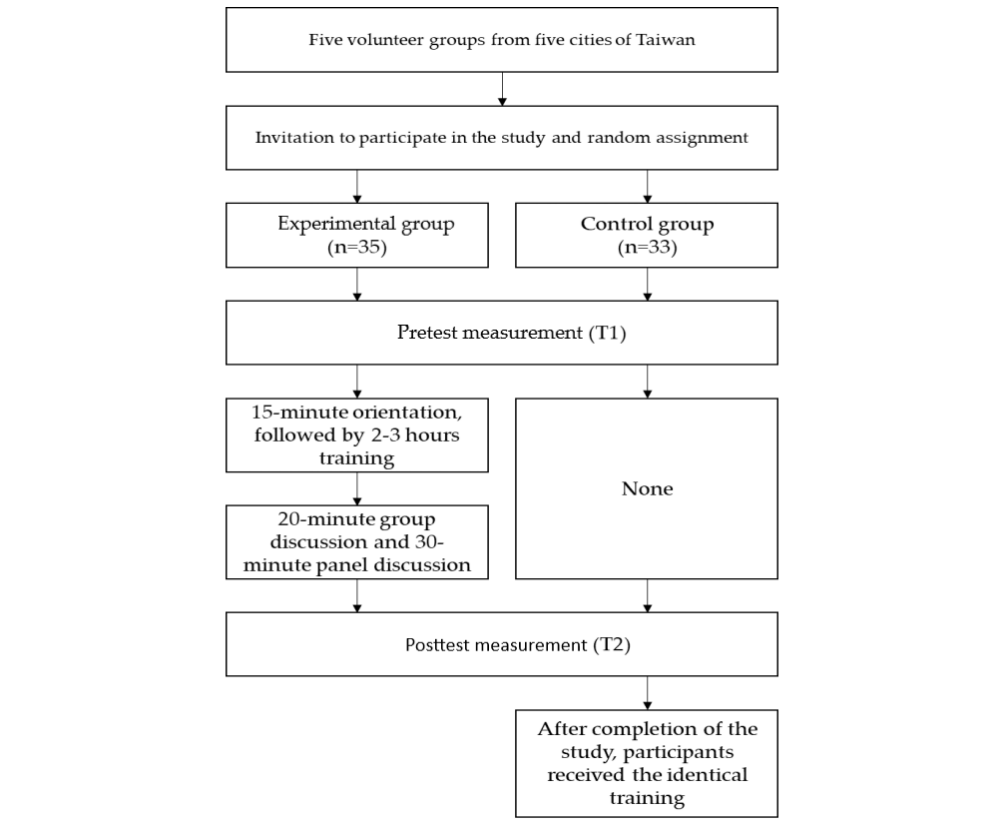
Flowchart showing the participant enrollment and assessment process.

A flowchart outlining participant enrollment and assessments is presented in Figure 1. After selecting the five Sunshine volunteer groups, the research team approached the executive director and staff to explain the research purpose, method, and protocol. After obtaining permission to conduct the study, we posted recruitment messages and held meetings to invite potential participants who met the inclusion criteria so that the participants could fully understand the purpose of the study before providing their written consent. After the meeting, we conducted a 15-minute operation session with SVVR to evaluate the feasibility and acceptance of the program. Participants indicated their acceptance of this arrangement and reported that the SVVR program is very novel and different from their previous training experiences. The research team members collected their baseline data in a quiet room of a local school. During the implementation period, a 3D VR technical professional, staff of the volunteer group, and several trainers of the SVVR program were available to ensure the training proceeded smoothly. After the SVVR training, participants of the experimental group were involved in a 20-minute group discussion. After the group discussion, a counseling professional hosted a 30-minute panel discussion to answer the questions regarding how to effectively help adolescents quit substance use. The 20-minute group discussion and 30-minute panel discussion were conducted to assist participants to organize what they learned into 5 VR scenarios and use them during the future accompanying process with students. After the panel discussion, the participants provided written and verbal feedback to the research team. The participants were encouraged to contact the counseling professional in future to work together in supporting students to quit using illegal drugs. The control group did not receive the SVVR training at the same time but received it after completion of the study.

### Training Program Development

**Figure 2:**
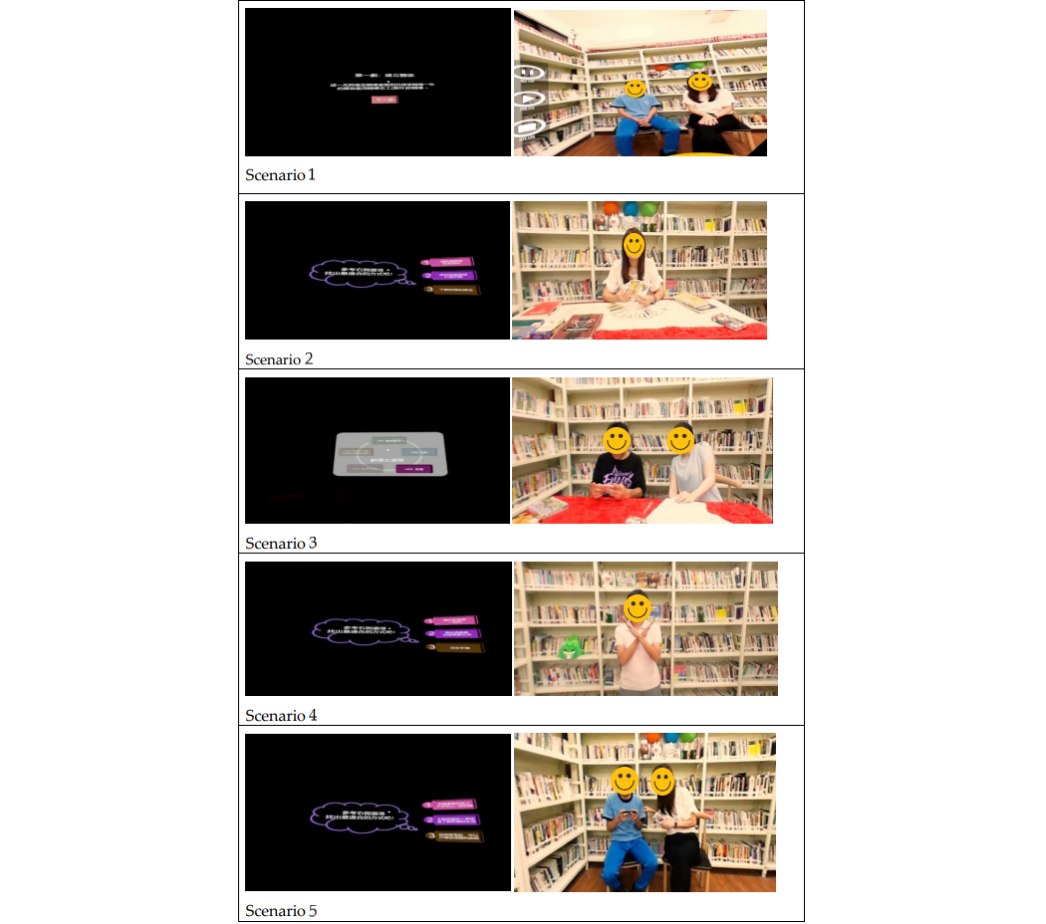
Five scenarios of spherical video-based virtual reality.

Before developing the SVVR program, the research team interviewed five volunteers face to face to understand how to help students quit substance use. Verbatim transcripts of the interviews were recorded. Consequently, the research team developed the SVVR program involving drug education, educational psychology and counseling, social work, nursing, and health education professionals to ensure the appropriateness of the SVVR program for the volunteers. The development of scenarios adopted a narrative approach in which each scenario included elements such as the setting, character, and plot. In addition, the context was derived from previous interviews of volunteers. This presentation format could make the scenarios more complete and better meet the needs of the volunteers. After the first draft of the training program was completed, the research team invited the volunteers and professionals to examine the five scenarios and provide their comments. The five scenarios are shown in Figure 2.

### Program Components

The training program offered 5 VR scenarios and invited participants to actively engage in a 20-minute group discussion and a 30-minute panel discussion. Table 1 summarizes the five scenarios. Each scenario has a corresponding activity led by a drug use prevention professional to guide participants in having proper interactions with students. For example, the first scenario focused on ice breaking, greeting, mutual introduction, and welcome messages. In the second scenario, students chose three Tarot cards and interpreted the meanings of these cards. The third scenario was about how to face a situation when students play mobile games and neglect the participants. The fourth scenario was to use Kahoot, a game-based learning platform, to design 10 questions for exploring ways of facilitating communication between students and close peers using drugs. The fifth scenario taught the participants how to become friends or followers of the students on social media platforms (Line, Facebook, and Instagram) to assess the social groups and lifestyles of the students, regularly or irregularly post positive messages, offer students timely attention, and interact with the students when needed. The relationships of the training program components and outcome variables are presented in Table 1.

The research team conducted a 15-minute operation demonstration to introduce the purpose of the training before the SVVR training was formally launched. In this orientation, the 3D VR professional taught the participants how to wear the 3D VR helmets and select the VR scenes. The participants practiced multiple times to avoid dizziness.

**Table 1:** Activities of virtual reality training program and outcome variables.

Activities for VR^a^ training program						VR1			VR2				VR3			VR4			VR5					
**Outcome variables**																								
	**Life skills**																							
		**Problem-solving (Information Children” [27]; Docktor and Heller [28])**																						
		Step 1: Define the problem.			Warm up: Welcome, introduce each other, explain that you are a volunteer who is happy to accompany the students and ask them about their recent life.					Tarot card game: Pick up the first card and pretend the card reveals the students’ current situation or problem. Participants interpret the card using information collected in advance.			N/A^b^			Help students identify if they have close peers (such as a buddy, boyfriend, or girlfriend) with drug use problems. When there is a binding relationship between the student and drug-using peers that prevents students from staying away, take the opportunity to advice students about the right way to interact with others.			N/A					
		Step 2: Gather information			Assist students in clarifying recent problems in life.			Tarot card game: Pick up the second card and encourage students to find the possible causes that resulted in the problems in their current lives.				N/A			N/A			N/A						
		Step 3: Generate possible solutions.			Express your concern, empathize, and invite students to brainstorm ideas about what has happened.			Tarot card game: Pick up the third card and suggest what should the student do in future to avoid similar problems.				N/A			N/A			N/A						
		Step 4: Evaluate possible methods and choose one.			N/A			N/A				Prepare yourself to handle students’ problems and emotions.			N/A			Learn how to use one social media platform that is currently used by the students for timely communication with students.						
		Step 5: Implement the method to solve the problem and evaluate.			N/A			N/A				N/A			N/A			Learn how to send messages and emoticons or emojis using the app to help students solve their problems.						
		**Self-efficacy empowerment**																						
			**Enactive self-mastery**																					
			Coaching				Discuss with the students about what can be done to avoid problems in life.				N/A			N/A			N/A							
			Participation	Encourage students to continue this companionship.			N/A				N/A			N/A			N/A							
			**Role modeling**																					
			Demonstration	N/A			Participants also draw three Tarot cards to explain how to face difficult situations, such as asking for help from others, finding available resources, and calmly thinking about solving problems.				A VR professional shares his or her own experience and explains how to deal with problems effectively.			N/A			A VR professional demonstrates how to interact with students using social media platforms.							
			Mentoring	Introduce your role as the student's mentor and your willingness to assist him or her for at least 3 months or even until graduation.			N/A				N/A			Guide students to uncover one or more questions regarding close peers with drug use problems. Politely ask them to examine their interaction with their peers and tell them to think independently about “the good and bad” about people, affairs, and things.			A professional uses his or her mobile phone to demonstrate how to successfully interact with students for a long period of time.							
			**Verbal persuasion**																					
			Stimulation	N/A			N/A				Challenge participants to think about solutions.			N/A			N/A							
			Rewards	N/A			Indicate the benefits of facing problems and solving them.				N/A			N/A			N/A							
		**Teamwork**																						
			Committing to the development of teamwork	N/A			N/A				Tell participants to listen to students and respect their ideas so that students are willing to work together with the volunteers.			N/A			N/A							
			Performing assignments that elicit teamwork	N/A			N/A				N/A			N/A			Perform assignments with participants that elicit teamwork.							
			Focusing on the process	N/A			N/A				N/A			N/A			Focus on the process of observing students’ social groups and lifestyles and provide positive feedback if the student makes any changes that reduce illegal drug use.							
			Providing meaningful feedback	N/A			N/A				N/A			Provide several useful suggestions to interact with close peers.			N/A							

^a^VR: virtual reality.

^b^N/A: Not applicable.

### Measurements

The sociodemographic variables of the participants assessed at baseline included their gender, age, the number of years completed as a Sunshine volunteer, marital status, employment, and the number of served students. The performance impact was assessed based on three variables including problem-solving skills, self-efficacy, and teamwork.

### Problem-solving Skills

The problem-solving ability scale was modified from a previously evaluated instrument [29] comprising 6 items and was scored on a Likert-type scale ranging from 1 to 5, with higher scores indicating a higher level of problem-solving abilities in terms of drug use prevention.

### Self-efficacy

The self-efficacy scale was a modified form of a previously tested scale [30] containing 6 items. Each item was scored on a Likert-type scale ranging from 1 to 5, with higher scores indicating a higher level of confidence in drug use prevention.

### Teamwork

The team cooperation scale used was a modified version of a previously tested scale [31] with 4 items. Each item was scored on a Likert-type scale ranging from 1 to 5, with higher scores indicating a higher level of cooperation with team members. Analyses of the Cronbach α and exploratory factor analysis of each variable indicated that a single factor on each scale accounted for 64.26% to 65.20% of the variance, as shown in Table 2.

**Table 2:** Reliability and factor loadings of each variable.

Theme	Number of items	Cronbach α	Factor	Factor loading	Accounted variance (%)
Problem-solving skills	6	.888	1	0.764-0.842	64.64
Self-efficacy	6	.887	1	0.699-0.882	64.26
Teamwork	4	.811	1	0.589-0.899	65.20

### Data Analysis

We conducted descriptive analysis of the demographic variables. In addition, two-tailed χ^2^ tests were conducted to compare differences based on the gender, age, number of years completed as a Sunshine volunteer, marital status, employment, and number of served students. A generalized estimating equation (GEE) was used to explore the effects of problem-solving, self-efficacy, and teamwork. The GEE enables the understanding of the changes and their effects at the individual and group levels by estimating the average response of the population and is more advantageous than regression analysis that would enable prediction of the effect [32]. Statistical analyses were conducted using SPSS 22.0 (IBM Corp).

### Ethics Statement

The study received approval from the Research Ethics Review Committee of National Taiwan Normal University (201805HS007).

## Results

### Demographic Data

Most of the participants in the experimental and control groups were female, aged 51 to 60 years and married. They had served as Sunshine volunteers for 7 or more years and were unemployed. The participants in the experimental and control groups had no statistically significant differences in terms of their demographic variables, as observed in Table 3. The participants in both groups were treated equivalently when comparing the intervention effects.

**Table 3:** Demographic data of participants (N=68).

Characteristic			Experimental group (n=35), n (%)		Control group (n=33), n (%)		χ^2^ (*df*)			*P* value	
**Gender**								1.019 (1)			.31
	Male	10 (28.57)		6 (18.18)							
	Female	25 (71.43)		27 (81.82)							
**Age (years)**								1.13 (2)			.57
	≤50	7 (20)		7 (21.21)							
	51-60	19 (54.29)		14 (42.42)							
	≥61	9 (25.71)		12 (36.36)							
**Marital status**								0.41 (1)			.61
	Married	34 (97.14)		31 (93.94)							
	Others	1 (2.86)		2 (6.06)							
**Employment history (years)**								0.262 (1)			.61
	≤6	13 (39.39)		11 (33.33)							
	≥7	20 (60.61)		22 (63.67)							
**Employment**								0.883 (1)			.35
	Yes	11 (31.42)		14 (42.42)							
	Others	24 (68.57)		19 (57.58)							
**Number of served students**								0.243 (1)			.62
	≤1	18 (51.43)		15 (45.45)							
	≥2	17 (48.57)		18 (54.55)							

### Improvements in Training Variables

**Figure 3:**
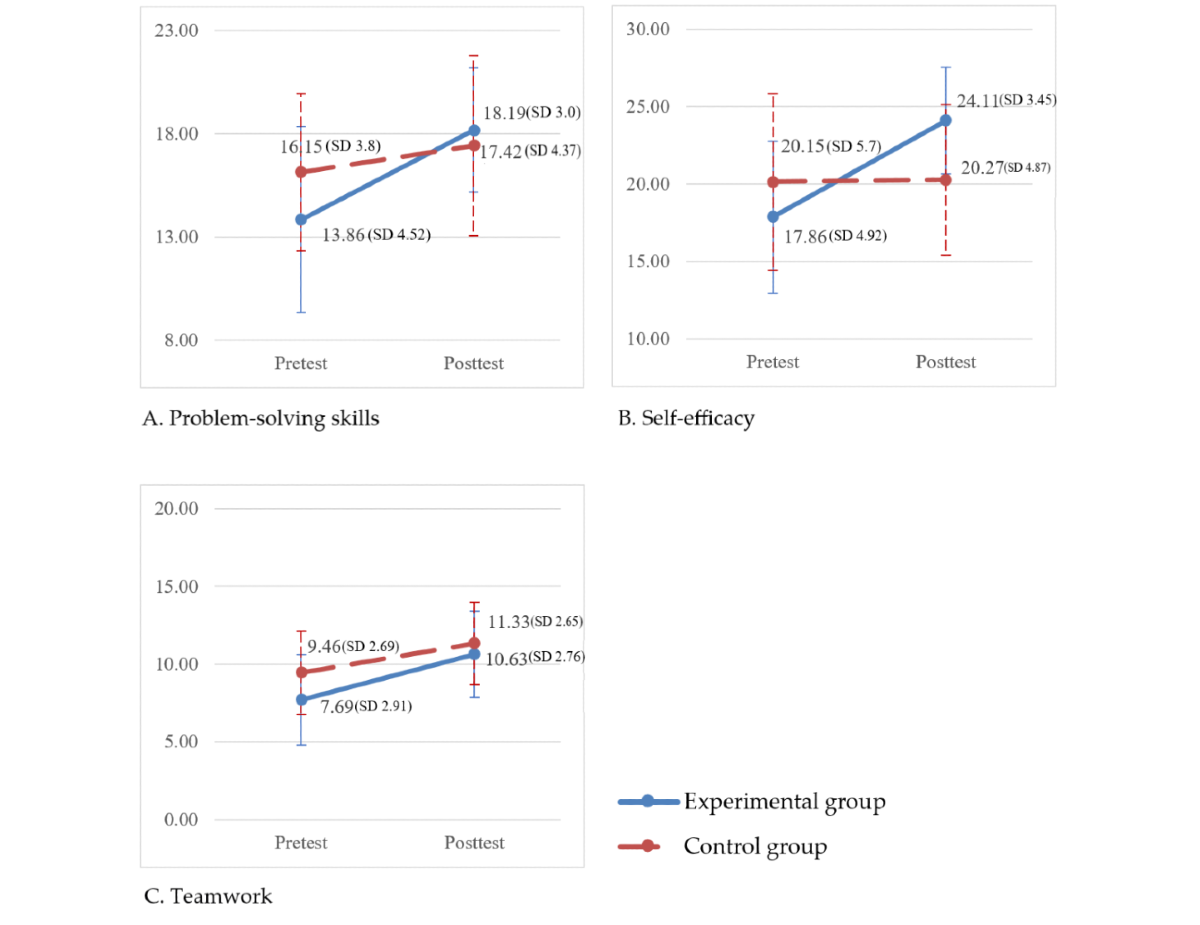
Changes in the experimental and control groups from pretest to posttest in terms of (A) problem-solving skills, (B) self-efficacy, and (C) teamwork.

Group differences in the patterns of change over time are shown in Figure 3. GEE analyses indicated a significant group × time interaction for problem-solving (β=3.055; Wald χ^2^_1_=4.757; *P*=.03) and self-efficacy (β=6.135; Wald χ^2^_1_=19.033; *P*<.001) but not for teamwork (β=1.025; Wald χ^2^_1_=1.172; *P*=0.28), as indicated in Table 4.

**Table 4:** Generalized estimating equation of outcome variables.

Life skill			Regression coefficient		SE	Wald χ^2^ (*df*)	*P* value
**Problem-solving**							
	Experimental group^a^	–2.283		1.107		4.250 (1)	*.04* ^b^
	Time (posttest)^c^	1.293		1.009		1.642 (1)	.20
	Experimental group × time (posttest)^d^	3.056		1.401		4.757 (1)	*.03*
**Self-efficacy**							
	Experimental group	–2.225		1.269		3.073 (1)	.08
	Time (posttest)^c^	0.295		0.998		0.087 (1)	.77
	Experimental group × time (posttest)	6.135		1.404		19.098 (1)	*<.001*
**Teamwork**							
	Experimental group	–1.708		0.730		5.471 (1)	*.02*
	Time (posttest)	1.880		0.468		16.112 (1)	*<.001*
	Experimental group × time (posttest)	1.025		0.947		1.172 (1)	*.28*

^a^reference group: control group

^b^Italicized values indicate statistical significance at *P*>.05

^c^reference group: baseline

^d^reference group: control × baseline

### Group and Panel Discussions

After the SVVR training, we invited the experimental group to discuss related responsive strategies. As shown in Table 5, the participants suggested responsive strategies including target setting, value identification, problem-solving skills, peer or parental influence, and environmental changes to assist students. During the panel discussion, the invited professionals and participants discussed responsive strategies including analyzing current situations, making multiple attempts, providing social support, and increasing social connections for students after quitting drugs considering their future life.

**Table 5:** Summary of responsive strategies for group and panel discussions.

Discussion	Responsive strategies
Group discussion: How do you help your students to avoid negative effects when drug-taking close peers still instill the wrong drug-taking concepts and ideas?	Target setting Support students to understand their interests and expertise. There are goals to divert students’ attention from drug use; the opportunities of success in discontinuing drug use are relatively high. (P1)a By understanding the family background, friends, and working conditions of the students, we can provide responsive suggestions and tell them how and what they can achieve in future so that the students find the courage to live a new life. (P2) Tell students that they will have more job opportunities if they possess a skill. (P1) Help them find the focus of their life (things they are interested in) and gradually get rid of drugs. (P4) Help students find the type of work they are interested in and explain that all their efforts in life will help them achieve something. (P5) Value identification Drugs harm health. For example, tell students that long-term use of ketamine will require them to use diapers for the rest of their life. (P9) Problem-solving First understand the causes of drug use, analyze the advantages and disadvantages of drug use, discover the problems, and then help students solve the problems and return to a healthy life. (P3) Inform the police about the drug supplier so that the students will not have access to drugs. (P12) Peer or parental influences Let students know that they can leave peers who use drugs, meet with groups engaged in healthy activities, and make good new friends to support and encourage each other. (P4) Cut off contacts with peers who use drugs. (P8) Delete the phone numbers of peers who use drugs from the students' mobile phones. (P12) Peers with risks of drug usage are also treated as persons needing care. (P12) Parents are critical of students who use drugs. They should listen to and engage with these students. Environmental change Choose the workplace carefully to avoid exposure to drugs. (P7) Choose extracurricular activities and do not participate in activities that expose students to drugs. (P7) Move out of the current living community to avoid exposure to drugs. (P8)
Panel discussion: Students ask what they can do if they quit drugs?	Analyzing current situations First understand the current situations of the students’ life and then provide specific suggestions. (P6) First explore students' interests, talk about their needs, guide them to think about future career planning, and then guide them to focus on their dreams. If they work hard, they can achieve all the good things they want. (P10) Multiple attempts Encourage students to participate in government-sponsored vocational training and explore more ways to establish their own career direction. (P1) Work in an interesting workplace to see if students are suitable for the job position. (P2) Forget the past and try to move forward. Keep the same job for at least 3 months before changing. (P2) Lead the students to participate in academic and industry collaborations. (P5) Help students find jobs. (P6) Introduce them to appropriate work opportunities according to their personality and expertise. (P9) Giving social support First address the students' emotions; let them take a break and let go of their blind spots; try to make them break through the bottleneck of life at this stage. (P5) Support students till the end and try to help them. (P7) Engage with students to find their interests; as long as they do not use drugs, everything is fine. (P8) Support students to make sensible decisions and be their backup. (P9) Get to know the students and help them. (P9) Let students work or start a business together; provide consultation and assistance in this process. (P13) Increasing social connections Encourage students to volunteer to care for the elderly and orphans and learn to help others and socialize. (P9)

^a^P: serial number of the participants.

## Discussion

### Principal Findings

To the best of our knowledge, this was the first study using SVVR in a volunteer training program. We adopted a randomized controlled design and recruited 68 participants from 5 cities to have enough power to detect the training effects. Although the score improvement aspects among the participants in the experimental group were superior to those shown by their counterparts in the control group in terms of problem-solving skills, self-efficacy, and teamwork after training, the GEE results revealed that the participants in the experimental group showed better improvements in problem-solving skills and self-efficacy compared with their counterparts in the control group but not in teamwork capabilities.

The SVVR training program improved the participants’ problem-solving abilities when compared to their counterparts in the control group. Problem-solving is one of the life skills that was advocated by the World Health Organization [33]. Problem-solving skills involve identifying a problem, developing possible solution paths, and taking the appropriate course of action. Problem-solving skills can improve adolescents’ problematic behaviors such as substance use [34]. The use of VR technology in the training program offers a novel opportunity for the development of problem-solving skills by providing learners with richer situations; this made the learning process more interesting and interactive and could improve the learners’ motivation and attention, thus helping them explore new possibilities. A recent study supported the use of VR technology stating that it could increase users’ interest and motivation, and potentially assist students in developing problem-solving skills [35]. Apart from the SVVR training program, our study conducted group and panel discussions. It contributed to the development of participants’ problem-solving skills through discussion processes so that the volunteers could understand the importance of having discussions with the other participants.

Another important finding of the study is that the SVVR training program was effective in promoting volunteers’ self-efficacy to help students quit drugs. Self-efficacy is one of the core concepts in Bandura's social learning theory, which refers to the degree of personal confidence in assessing whether a particular behavior is performed in a specific situation. It is a critical variable in predicting and interpreting future behavior [10]. A previous study pointed out that self-efficacy can stimulate behaviors and is the source of motivation [36]. The higher the self-efficacy, the higher is the number of expected healthy behaviors [37]. Self-efficacy could be a significant predictor for drug quitting among adolescents [5]. In addition, self-efficacy reflects the proficiency in personal abilities. An individual’s self-efficacy can be improved through personal experiences of success [38]. Our findings advocate the use of SVVR for improving participants’ self-efficacy, and they are consistent with the findings of previous studies [39,40]. A recent study indicated that using VR-based learning environments with student teachers helped students increase their self-efficacy and allowed them to be more innovative and creative [41]. Thus, using VR for training adolescents in self-efficacy offers high potential at present and in future.

A previous study identified significant differences between virtual and live simulations in terms of teamwork attitudes and communication skills in a randomized controlled trial that was conducted with 120 undergraduate medical and nursing students. These findings supported the potential of VR as a substitute for conventional team-based simulation training [42]. Numerous learning and performing tasks require teamwork. Team members may work concurrently and meet during some occasions. A recent review article summarizing trainings for health professionals indicated that most review studies evaluated the usability and acceptability of VR simulations whereas very few studies have measured the effects of VR simulations on the development of nontechnical skills such as teamwork [43]. Researchers indicated that although the importance of teamwork in health care is recognized, limited consensus exists regarding what it is, how it can most effectively be learned, and how it should be assessed [44]. We suggest that collaborative virtual environments should be developed in future training programs to allow participants to come together to complete a task. VR might have different educational or training effects on teamwork, suggesting that more research is needed in future to clarify its effectiveness.

### Conclusions

We conclude that it is feasible to apply 3D VR technology in training volunteers who help with substance use prevention. The results of this study revealed that SVVR can improve participants’ problem-solving and self-efficacy for assisting students in quitting illegal drug use. However, future studies are suggested to develop effective SVVRs to assist inexperienced volunteers in enhancing their teamwork abilities. We believed that it is promising to introduce this training program at more sites to enhance volunteer training so that volunteers can be better companions and help students quit drugs.

## Appendix

Multimedia Appendix 1 CONSORT-eHEALTH checklist (V 1.6.1).

## Abbreviations

SVVR: spherical video-based virtual reality
VR: virtual reality
